# Non-hematopoietic roles of erythropoietin in inflammation and metabolic disorders

**DOI:** 10.3389/fphar.2025.1613849

**Published:** 2025-07-08

**Authors:** Satoru Sugimoto, Takeshi Goda

**Affiliations:** Department of Pediatrics, Graduate School of Medical Science, Kyoto Prefectural University of Medicine, Kyoto, Japan

**Keywords:** erythropoietin, obesity, inflammation, liver, white adipose tissue, non-hematopoietic EPO analogs

## Abstract

Erythropoietin (EPO), a glycoprotein hormone primarily produced by the kidneys, is essential for erythropoiesis. Beyond its well-established hematopoietic function, EPO has emerged as a regulator of metabolic inflammation. Obesity-induced chronic inflammation underlies insulin resistance, a key driver of metabolic disorders such as type 2 diabetes mellitus (T2DM) and metabolic dysfunction-associated steatohepatitis (MASH). Recent evidence shows EPO exerts anti-inflammatory effects in insulin-sensitive tissues, thereby improving insulin sensitivity in the context of obesity. Although EPO is clinically approved for treating anemia in both neonates and adults, further evaluation is needed to establish its safety and tolerability when repurposed for metabolic indications across these populations. Nevertheless, to overcome the hematopoietic side effects of native EPO, researchers have developed non-hematopoietic analogs with selective tissue-protective actions. These analogs are currently under investigation and have shown therapeutic potential without erythropoietic side effects. This review summarizes the anti-inflammatory roles of EPO in obesity-related metabolic dysfunction, particularly in white adipose tissue (WAT) and the liver, and discusses the therapeutic potential of non-hematopoietic EPO analogs.

## Introduction

Erythropoietin (EPO) is a hypoxia-inducible glycoprotein hormone mainly synthesized in the kidneys, known for promoting red blood cell production. Clinically, it is widely used to treat anemia associated with chronic kidney disease and prematurity. However, EPO receptors (EPORs) are also expressed in non-hematopoietic tissues—including the brain, heart, liver, adipose tissue, and pancreas—indicating broader biological roles in neuroprotection, angiogenesis, metabolic regulation (Allwood et al., 2024; Liu Q. S. et al., 2015; Shuai et al., 2011; Vittori et al., 2021; Yin et al., 2024). EPOR expression has also been observed in immune cells such as macrophages, T cells, and dendritic cells, suggesting a direct immunomodulatory function (Liu et al., 2023; Chen et al., 2020; Peng et al., 2020). With the progression of obesity, immune cells including macrophages and neutrophils infiltrate insulin-sensitive tissues such as white adipose tissue (WAT), the liver, and skeletal muscle. This chronic inflammation impairs insulin signaling and drives metabolic disorders including type 2 diabetes mellitus (T2DM), hypertension, atherosclerosis, and metabolic dysfunction-associated steatotic liver disease (MASLD), formerly known as nonalcoholic fatty liver disease (NAFLD) (Peng et al., 2020). Given the intricate crosstalk between inflammation and metabolic dysfunction, considerable research efforts have been directed toward therapeutic strategies targeting inflammatory pathways in insulin-sensitive tissues (Mak, 1998; Kodo et al., 2017; Tsuma et al., 2019; Teng et al., 2011; Wang et al., 2013; Foskett et al., 2011).

Recent studies indicate that EPO exerts potent anti-inflammatory effects across multiple tissues, including the central nervous system, cardiovascular system, and metabolic organs. In insulin-sensitive tissues, particularly WAT and the liver, EPO plays a critical role in immune homeostasis. This review focuses on the actions of EPO in WAT and the liver, as most studies examining EPO in the context of obesity-associated inflammation in insulin-sensitive organs have concentrated on these two metabolic tissues. Relevant literature published between 2010 and 2025 was identified through searches of PubMed and Google Scholar using keywords such as “erythropoietin,” “obesity,” “adipose tissue,” “liver,” “inflammation,” and “nonhematopoietic analogs.” One seminal publication prior to 2010 was also included due to its particular relevance.

## EPO and WAT inflammation in obesity

WAT inflammation is a key feature of obesity-induced insulin resistance. To date, two studies have examined the effects of EPO on WAT inflammation (Alnaeeli et al., 2014; Pan et al., 2013), and one has evaluated a non-hematopoietic EPO analog (Liu Y. et al., 2015). These three studies are highlighted here as they provide valuable insights into the relationship between EPO and WAT inflammation in obesity, representing the most informative data currently available.

Alnaeeli et al. (2014) demonstrated that EPO improves insulin sensitivity and glucose tolerance in diet-induced obese (DIO) mice without affecting body weight or perigonadal fat mass, effects closely linked to reduced inflammation in WAT. EPOR was found to be highly expressed in the stromal vascular fraction (SVF) of perigonadal WAT, particularly in macrophages, whereas its expression in skeletal muscle was minimal. EPO administration led to decreased macrophage infiltration in WAT, as demonstrated by immunofluorescence and flow cytometry, alongside a shift in circulating cytokine profile: reduced tumor necrosis factor-alpha (TNF-α) and increased interleukin-10 (IL-10) levels. They also demonstrated that EPO administration expands anti-inflammatory macrophages through a mechanism dependent on the interleukin-4/signal transducer and activator of transcription 6 (IL-4/STAT6) signaling axis, suggesting that EPO modulates not only macrophage number but also phenotype in WAT. Obese EPOR-deficient mice with erythroid-specific EPOR expression exhibited impaired glucose tolerance and insulin resistance, despite having body weight and fat mass comparable to wild-type controls. In the EPOR-deficient mice, WAT showed increased macrophage infiltration, with a predominance of pro-inflammatory over anti-inflammatory subtypes. Thus, these findings suggest that EPO acts directly on immune cells within WAT to modulate inflammation independent of changes in adiposity.

Pan et al. (2013) investigated EPO’s direct effects on adipocytes using 3T3-L1 cells, a well-established model for studying mechanisms of obesity and related pathologies. They found that EPOR expression increased markedly during adipocyte maturation, suggesting an active role for EPO in mature adipocytes. Insulin resistance was modeled by treating mature 3T3-L1 adipocytes with dexamethasone, which impaired glucose uptake in a dose-dependent manner. EPO restored glucose uptake in these insulin-resistant adipocytes *via* an EPOR-dependent mechanism, without affecting cell proliferation. EPO also increased AKT expression in insulin-resistant mature adipocytes, an effect that was antagonized by a phosphoinositide 3-kinase (PI3K) inhibitor, suggesting involvement of the EPOR/PI3K/AKT signaling axis. Additionally, EPO dose-dependently suppressed proinflammatory adipokines TNF-α and leptin, while enhancing adiponectin secretion. EPO increased STAT5 expression in insulin-resistant adipocytes, an effect abrogated by a STAT5 inhibitor, implicating EPOR/JAK/STAT5 upregulation in its anti-inflammatory action. Collectively, these findings suggest that, in adipocytes, EPO improves the inflammatory profile and attenuates insulin resistance *via* non-canonical insulin signaling mechanisms.

Following the above two studies, Liu Y. et al. (2015) investigated the anti-inflammatory and metabolic effects of pHBSP—an engineered, non-hematopoietic EPO-derived peptide that selectively activates the tissue-protective receptor (TPR) without inducing erythropoiesis—using both *in vivo* and *in vitro* models. They demonstrated that pHBSP improved insulin sensitivity and lipid profiles in DIO mice, without stimulating erythropoiesis. In epididymal WAT (eWAT) of DIO mice, pHBSP treatment markedly reduced the expression of pro-inflammatory cytokines, including TNF-α, interleukin-6 (IL-6), and inducible nitric oxide synthase (iNOS) and increased the expression of anti-inflammatory markers, such as found in inflammatory zone 1 (Fizz-1) and arginase-1, not only in whole tissue but also within the SVF. In addition, pHBSP decreased macrophage infiltration, as evidenced by a reduced number of crown-like structures in eWAT. Furthermore, pHBSP treatment led to a reduction in eWAT mass in DIO mice. In support of this finding, *in vitro* experiments showed that pHBSP suppressed lipid accumulation and inhibited adipocyte differentiation in 3T3-L1 cells in a dose-dependent manner, accompanied by downregulation of peroxisome proliferator-activated receptor gamma (PPARγ) expression. pHBSP also significantly reduced the expression of TNF-α, IL-6, and monocyte chemoattractant protein-1 (MCP-1) in 3T3-L1 adipocytes through TPR-dependent mechanisms. These results suggest that the anti-inflammatory effects of pHBSP on adipocytes may be mediated not only by direct molecular actions but also by secondary effects resulting from reduced adiposity. Additionally, they found that pHBSP suppressed the expression of TNF-α and IL-6 in LPS-stimulated macrophage cultures in a TPR-dependent manner. Collectively, these findings indicate that pHBSP exerts direct anti-inflammatory actions on both immune cells and adipocytes within WAT, and that its modulation of adipocyte lipid metabolism may partially contribute to anti-inflammatory effects.

Together, these three studies underscore that both classical erythropoietin and its non-hematopoietic derivatives can modulate inflammation and insulin sensitivity through distinct molecular mechanisms, positioning WAT as one of the key targets of EPO-based metabolic regulation.

## EPO and hepatic inflammation in obesity

Hepatic steatosis is a highly prevalent complication of obesity and represents a key step in the progression toward more severe liver pathologies, including fibrosis, cirrhosis, and hepatocellular carcinoma. Chronic inflammation is a critical driver in the transition from simple steatosis to these advanced stages and contributes to systemic metabolic dysfunction. Despite its clinical burden, there are currently no approved pharmacologic therapies that directly target the underlying mechanisms of fatty liver disease, whether referred to under the traditional nomenclature of nonalcoholic fatty liver disease (NAFLD) and its progressive form, nonalcoholic steatohepatitis (NASH), or the newer terminology of metabolic dysfunction-associated fatty liver disease (MAFLD) and steatohepatitis (MASH), underscoring the urgent need for effective treatment strategies (Anstee et al., 2013; Younossi et al., 2016).

Experimental data suggest that EPO exerts beneficial effects on hepatic inflammation and glucose metabolism in obesity. Meng et al. (2013) identified EPOR mRNA transcription in the liver, indicating that the liver may be a direct target of EPO. In the liver of DIO mice, EPO administration downregulated the expression of Toll-like receptor 4 (TLR4) and suppressed downstream activation of Nuclear Factor kappa-light-chain-enhancer of activated B cells (NF-κB) and c-Jun N-terminal kinase (JNK), leading to reduced levels of TNF-α and IL-6. These anti-inflammatory effects were accompanied by improved insulin sensitivity, independent of changes in food intake. In the study, EPO also activated the PI3K/AKT pathway without altering upstream insulin receptor (IR) or insulin receptor substrate 1 (IRS1) phosphorylation, and reduced the expression of key gluconeogenic enzymes, including phosphoenolpyruvate carboxykinase (PEPCK) and glucose-6-phosphatase (G6Pase) in the liver, suggesting that EPO may enhance hepatic glucose metabolism through a non-canonical insulin signaling pathway. Additional mechanistic insight comes from *in vitro* studies. Zhang et al. demonstrated that in palmitic acid-induced insulin-resistant hepatocytes, EPO improved insulin signaling by activating the IRS/AKT/FOXO1 (Forkhead box protein O1) and phosphorylated glycogen synthase kinase 3β (p-GSK-3β) pathways, leading to increased glycogen content. These effects were accompanied by a reduction in the expression of proinflammatory cytokines, including MCP-1, TNF-α and interleukin-1β (IL-1β) (Zhang et al., 2017). Although inflammatory markers were not assessed, Ge et al. reported that EPO enhanced hepatic insulin signaling through a PPARγ-dependent PI3K/AKT axis, involving upstream Ca2+/calmodulin-dependent kinase (CaMKK), AMP-activated protein kinase (AMPK), and sirtuin 1 (SIRT1) (Ge et al., 2015). Taken together, these *in vivo* and *in vitro* experiment findings support a potential role for EPO in reducing hepatic inflammation, though the molecular pathways appear to vary across experimental models.

Further support for EPO’s hepatic actions stems from studies outside the context of obesity. EPO has been shown to directly modulate Kupffer cell activity, the liver-resident macrophage population (Gilboa et al., 2017), and preliminary clinical data suggested that EPO, in combination with granulocyte colony-stimulating factor, may improve outcomes in patients with decompensated cirrhosis (Kedarisetty et al., 2015). Although these studies are not directly focused on metabolic liver disease, they reinforce the plausibility of EPO-mediated immunomodulation in hepatic tissue.

In sum, current studies strongly suggest that EPO exerts direct beneficial effects on hepatic inflammation, thereby improving glucose metabolism in obesity. As with its anti-inflammatory actions in WAT, however, the molecular pathways through which EPO modulates hepatic inflammation appear to vary across studies.

## Non-hematopoietic EPO analogs in human studies

The classical EPO molecule is unlikely to be suitable for clinical application due to its erythropoietic side effects, including polycythemia, thrombotic events, and hypertension. To address this, non-hematopoietic EPO-derived peptides have been developed. Among these, ARA 290 has shown clinical promise. In a phase 2 trial involving patients with T2DM and painful neuropathy, ARA 290 improved glycemic control and lipid profiles without significant adverse effects (Brines et al., 2015). It has also demonstrated benefit in other inflammatory conditions, such as sarcoidosis-associated small fiber neuropathy (Culver et al., 2017; Dahan et al., 2013). Although evidence in obesity-related inflammation remains limited, these findings support the therapeutic potential of non-hematopoietic EPO analogs.

## Discussion

This review has highlighted the role of EPO in modulating inflammation within WAT and the liver, thereby enhancing insulin sensitivity and contributing to metabolic regulation. While EPO demonstrates clear metabolic benefits in preclinical models, its erythropoietic activity poses clinical limitations—particularly in individuals with obesity-related comorbidities—due to risks such as polycythemia and thrombosis. Consequently, non-hematopoietic EPO analogs have emerged as promising therapeutic alternatives. Robust evidence from animal and cellular studies supports the anti-inflammatory and metabolic effects of EPO; however, clinical data on non-hematopoietic EPO analogs in patients with obesity and metabolic disorders remain limited. Rigorous clinical trials are therefore warranted to establish their safety and efficacy across diverse populations. In addition, investigating potential synergy with lifestyle interventions or established pharmacotherapies, such as glucagon-like peptide-1 (GLP-1) receptor agonists, may further inform their therapeutic utility.

In conclusion, although differing in molecular mechanisms, EPO exerts anti-inflammatory and metabolic actions in key insulin-sensitive tissues, positioning it as a compelling target in obesity-associated metabolic disorders. Advancing the development and clinical validation of safe, non-hematopoietic EPO analogs is essential for translating these findings into clinical application. Future studies should also assess their relevance in other insulin resistance–related conditions, including cardiovascular disease and polycystic ovary syndrome, and define their optimal therapeutic indications.
